# A tetra­gonal polymorph of bis­[hydro­tris­(pyrazol-1-yl)borato]iron(II)

**DOI:** 10.1107/S1600536811025839

**Published:** 2011-07-06

**Authors:** Zhong-Hai Ni, Guo-Ling Li, Rui Ma, Jing Nie

**Affiliations:** a School of Chemical Engineering and Technology, China University of Mining and Technology, Xuzhou 221116, Jiangsu Province, People’s Republic of China

## Abstract

The title compound, [Fe(C_9_H_10_BN_6_)_2_], is a polymorph of a compound reported previously [Oliver *et al.* (1980 ▶). *Inorg. Chem.* **19**, 165–168]. In the previous report, the compound crystallized in the monoclinic space group *P*2_1_/*c* (*Z* = 4), whereas the crystal symmetry of the compound reported here is tetra­gonal (*P*4_2_/*ncm*, *Z* = 4). The mol­ecular structure is comprised of two hydro­tris­(1-pyrazol­yl)borate ligands (Tp^−^) and a central Fe^II^ ion, which is coordinated by six pyrazole N atoms from two two Tp^−^ ligands, yielding a distorted bipyramidal FeN_6_ geometry. The complete molecule exhibits symmetry 2/*m*.

## Related literature

For the crystal structure of the other polymorph measured at room temperature, see: Oliver *et al.* (1980 ▶). For iron(II) complexes with the Tp^−^ derivative ligands, see: Janiak *et al.* (2000 ▶); Reger *et al.* (2005 ▶). 
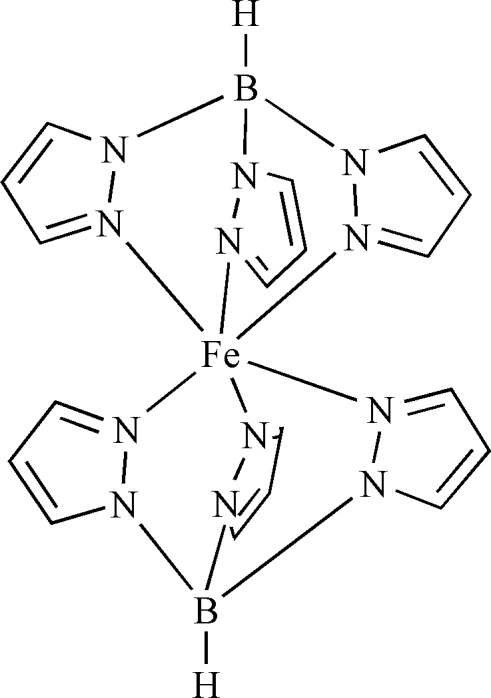


## Experimental

### 

#### Crystal data

[Fe(C_9_H_10_BN_6_)_2_]
*M* *_r_* = 481.93
Tetragonal, 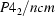

*a* = 17.017 (3) Å
*c* = 7.4099 (15) Å
*V* = 2145.7 (7) Å^3^
*Z* = 4
Mo *K*α radiation
μ = 0.74 mm^−1^
*T* = 123 K
0.20 × 0.15 × 0.12 mm

#### Data collection

Bruker APEXII CCD area-detector diffractometer
Absorption correction: multi-scan (*SADABS*; Sheldrick, 2003 ▶) *T*_min_ = 0.867, *T*_max_ = 0.916
14091 measured reflections
1099 independent reflections
1095 reflections with *I* > 2σ(*I*)
*R*_int_ = 0.034

#### Refinement

*R*[*F* ^2^ > 2σ(*F* ^2^)] = 0.037
*wR*(*F* ^2^) = 0.123
*S* = 0.95
1099 reflections
87 parameters
H-atom parameters not refined
Δρ_max_ = 0.43 e Å^−3^
Δρ_min_ = −0.39 e Å^−3^

### 

Data collection: *APEX2* (Bruker, 2004 ▶); cell refinement: *SAINT-Plus* (Bruker, 2001 ▶); data reduction: *SAINT-Plus*; program(s) used to solve structure: *SHELXS97* (Sheldrick, 2008 ▶); program(s) used to refine structure: *SHELXL97* (Sheldrick, 2008 ▶); molecular graphics: *XP* (Sheldrick, 1998 ▶); software used to prepare material for publication: *XP*.

## Supplementary Material

Crystal structure: contains datablock(s) global, I. DOI: 10.1107/S1600536811025839/hg5058sup1.cif

Structure factors: contains datablock(s) I. DOI: 10.1107/S1600536811025839/hg5058Isup2.hkl

Additional supplementary materials:  crystallographic information; 3D view; checkCIF report

## Figures and Tables

**Fig. 1.**
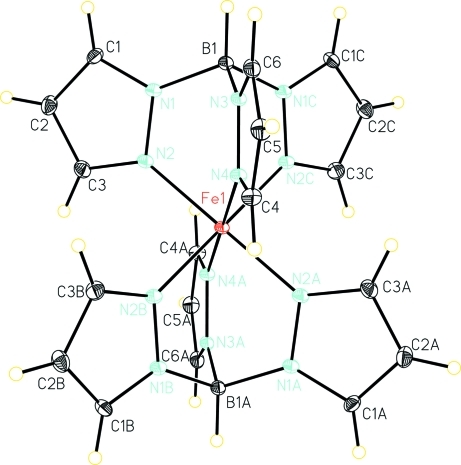
A view of (I) with the unique atom-labelling scheme. Displacement ellipsoids are drawn at the 30% probability level. [symmetry code: (A) -x, -y, -z; (B) -y, -x, -z; (C) +y, +x, +z.]

## supplementary crystallographic information

### Comment

Recently, hydro-tris(1-pyrazolyl)-borate (Tp^-^)and its derivatives have been employed as tridentate ligands to assembly molecular functional materials such as cyanide-bridged magnetic complexes, spin cross-over compounds and optic materials. In these cases, some mononuclear iron(II) complexes with two such tridendate ligands have been synthesized and crystal structures characterized (Janiak *et al.*, 2000; Reger *et al.*, 2005). The crystal structure of the title compound has been reported previously (Oliver *et al.*, 1980) which was measured at room temperature and crystallized in monoclinic space group of *P*2_1_/c (*Z* = 4). Recently, we synthesized this compound and measured its crystal structure at temperature 123 K. The result indicated that the crystal structure of the compound is significantly from the previous report. Herein, we report the crystal structure of the title compound [Fe^II^(C_9_H_10_N_6_B)_2_] (I).

The title compound in this paper crystallizes in a tetragonal space group *P*4_2_/ncm, suggesting there is a fourfold rotation symmetry axis in the unit cell. In the molecular structure of the title compound, there is a pseudo C_3_ rotation axis. The geometry and labeling scheme for the crystal structure of the title complex are depicted in Figure 1. The molecular structure of title compound in this work comprises of two Tp^-^ ligands and one central iron(II) ion. In the molecular structure, the cental metal iron(II) ion is coordinated by six pyrazole nitrogen atoms from the same two Tp^-^ ligands, yielding a distorted bipyramidal FeN_6_ geometry.

The Fe—N bond length is 1.975 (2)Å for Fe1—N2 and 1.983 (2)Å for Fe1—N4, respectively. which are simiar to those in the polymorph of the title compound reported previously at room temperature (Oliver *et al.*, 1980). The N—Fe—N bond angle is 89.05 (18)° for N2—Fe1—N4 and 88.33 (18)° for N2—Fe1—N2[+*y*, +*x*, +*z*.], respectively. These Fe—N bond lengthes suggest that the iron(II) center of the title compound is low spin state whether at low temperature or at room temperature (Oliver *et al.*, 1980).

### Experimental

The title complex was prepared as following: methanol solution (10 ml) of [Fe^II^(BF_4_)_2_].4H_2_O (30 mg, 0.1 mmol) was added slowly into a MeOH and aqueous solution (20 ml, water and methanol with *v*/*v* = 1/1) containing the ligand KTp (50.4 mg, 0.2 mmol). Then, the mixture was carefully filtered and the resulting solution was kept at room temperature for about two days, producing block brown crystals of (I) with yield 50%.

### Refinement

The coordinates of the H atom bound to boron atom was found from difference Fourier maps and refined freely. H atoms bound to C atoms were placed using the HFIX commands in *SHELXL-97*, with C—H distances of 0.93 Å. All H atoms were allowed for as riding atoms with *U*_iso_(H) = 1.2*U*_eq_(C).

### Crystal data

**Table:**

[Fe(C_9_H_10_BN_6_)_2_]	*D*_x_ = 1.492 Mg m^−^^3^
*M**_r_* = 481.93	Mo *K*α radiation, λ = 0.71073 Å
Tetragonal, *P*4_2_/*n**c**m*	Cell parameters from 1210 reflections
Hall symbol: -P 4ac 2ac	θ = 2.4–27.1°
*a* = 17.017 (3) Å	µ = 0.74 mm^−^^1^
*c* = 7.4099 (15) Å	*T* = 123 K
*V* = 2145.7 (7) Å^3^	Block, brown
*Z* = 4	0.2 × 0.15 × 0.12 mm
*F*(000) = 992	

### Data collection

**Table:**

Bruker APEXII CCD area-detector diffractometer	1099 independent reflections
Radiation source: fine-focus sealed tube	1095 reflections with *I* > 2σ(*I*)
graphite	*R*_int_ = 0.034
φ and ω scans	θ_max_ = 26.0°, θ_min_ = 3.4°
Absorption correction: multi-scan (*SADABS*; Sheldrick, 2003)	*h* = −20→20
*T*_min_ = 0.867, *T*_max_ = 0.916	*k* = −20→16
14091 measured reflections	*l* = −9→8

### Refinement

**Table:**

Refinement on *F*^2^	Primary atom site location: structure-invariant direct methods
Least-squares matrix: full	Secondary atom site location: difference Fourier map
*R*[*F*^2^ > 2σ(*F*^2^)] = 0.037	Hydrogen site location: inferred from neighbouring sites
*wR*(*F*^2^) = 0.123	H-atom parameters not refined
*S* = 0.95	*w* = 1/[σ^2^(*F*_o_^2^) + (0.0908*P*)^2^ + 2.7074*P*] where *P* = (*F*_o_^2^ + 2*F*_c_^2^)/3
1099 reflections	(Δ/σ)_max_ < 0.001
87 parameters	Δρ_max_ = 0.43 e Å^−^^3^
0 restraints	Δρ_min_ = −0.39 e Å^−^^3^

### Special details

**Table:**

Geometry. All e.s.d.'s (except the e.s.d. in the dihedral angle between two l.s. planes) are estimated using the full covariance matrix. The cell e.s.d.'s are taken into account individually in the estimation of e.s.d.'s in distances, angles and torsion angles; correlations between e.s.d.'s in cell parameters are only used when they are defined by crystal symmetry. An approximate (isotropic) treatment of cell e.s.d.'s is used for estimating e.s.d.'s involving l.s. planes.
Refinement. Refinement of *F*^2^ against ALL reflections. The weighted *R*-factor *wR* and goodness of fit *S* are based on *F*^2^, conventional *R*-factors *R* are based on *F*, with *F* set to zero for negative *F*^2^. The threshold expression of *F*^2^ > σ(*F*^2^) is used only for calculating *R*-factors(gt) *etc*. and is not relevant to the choice of reflections for refinement. *R*-factors based on *F*^2^ are statistically about twice as large as those based on *F*, and *R*- factors based on ALL data will be even larger.

### Fractional atomic coordinates and isotropic or equivalent isotropic displacement parameters (Å^2^)

**Table:**

	*x*	*y*	*z*	*U*_iso_*/*U*_eq_	
Fe1	0	0	0	0.0125 (3)	
B1	0.11871 (13)	0.11871 (13)	0.1589 (4)	0.0150 (6)	
H1A	0.1588 (14)	0.1588 (14)	0.211 (4)	0.013 (7)*	
C1	0.19598 (12)	0.00348 (11)	0.3210 (3)	0.0173 (5)	
H1	0.2365	0.0317	0.3749	0.021*	
C2	0.18477 (12)	−0.07653 (13)	0.3322 (3)	0.0218 (5)	
H2	0.2154	−0.1127	0.3947	0.026*	
C3	0.11737 (12)	−0.09163 (12)	0.2293 (3)	0.0201 (5)	
H3	0.0954	−0.1411	0.2117	0.024*	
C4	0.08225 (9)	0.08225 (9)	−0.3162 (3)	0.0176 (6)	
H4	0.0562	0.0562	−0.4090	0.021*	
C5	0.13997 (9)	0.13996 (9)	−0.3405 (3)	0.0196 (6)	
H5	0.1594	0.1594	−0.4491	0.024*	
C6	0.16160 (12)	0.16160 (12)	−0.1690 (4)	0.0179 (6)	
H6	0.1992	0.1992	−0.1401	0.021*	
N1	0.13791 (9)	0.03379 (9)	0.2180 (2)	0.0157 (4)	
N2	0.08928 (10)	−0.02509 (10)	0.1603 (2)	0.0162 (4)	
N3	0.11954 (9)	0.11954 (9)	−0.0499 (3)	0.0148 (5)	
N4	0.07002 (9)	0.07002 (9)	−0.1405 (3)	0.0152 (5)	

### Atomic displacement parameters (Å^2^)

**Table:**

	*U*^11^	*U*^22^	*U*^33^	*U*^12^	*U*^13^	*U*^23^
Fe1	0.0106 (3)	0.0106 (3)	0.0163 (4)	−0.00276 (17)	−0.00002 (13)	−0.00002 (13)
B1	0.0120 (9)	0.0120 (9)	0.0212 (15)	−0.0015 (11)	−0.0007 (8)	−0.0007 (8)
C1	0.0121 (10)	0.0198 (11)	0.0201 (10)	−0.0007 (7)	−0.0028 (8)	0.0015 (7)
C2	0.0177 (10)	0.0192 (10)	0.0283 (11)	0.0014 (8)	−0.0013 (8)	0.0071 (8)
C3	0.0198 (10)	0.0135 (9)	0.0270 (11)	−0.0020 (7)	0.0006 (8)	0.0030 (8)
C4	0.0183 (9)	0.0183 (9)	0.0162 (13)	−0.0024 (11)	−0.0005 (7)	−0.0005 (7)
C5	0.0194 (9)	0.0194 (9)	0.0201 (14)	−0.0011 (11)	0.0032 (8)	0.0032 (8)
C6	0.0136 (8)	0.0136 (8)	0.0266 (15)	−0.0008 (10)	0.0027 (8)	0.0027 (8)
N1	0.0144 (8)	0.0131 (9)	0.0197 (8)	−0.0031 (6)	−0.0012 (6)	−0.0007 (6)
N2	0.0144 (8)	0.0128 (8)	0.0216 (8)	−0.0034 (6)	−0.0004 (6)	−0.0009 (6)
N3	0.0111 (7)	0.0111 (7)	0.0223 (12)	−0.0020 (8)	0.0001 (6)	0.0001 (6)
N4	0.0134 (7)	0.0134 (7)	0.0190 (11)	−0.0024 (9)	−0.0006 (6)	−0.0006 (6)

### Geometric parameters (Å, °)

**Table:**

Fe1—N2^i^	1.9751 (17)	C2—C3	1.401 (3)
Fe1—N2^ii^	1.9751 (17)	C2—H2	0.9300
Fe1—N2	1.9751 (17)	C3—N2	1.331 (3)
Fe1—N2^iii^	1.9751 (17)	C3—H3	0.9300
Fe1—N4^iii^	1.981 (2)	C4—N4	1.335 (3)
Fe1—N4	1.981 (2)	C4—C5	1.4005
B1—N1^ii^	1.545 (2)	C4—H4	0.9300
B1—N1	1.545 (2)	C5—C6	1.374 (4)
B1—N3	1.547 (4)	C5—H5	0.9300
B1—H1A	1.04 (3)	C6—N3	1.343 (4)
C1—N1	1.351 (3)	C6—H6	0.9300
C1—C2	1.377 (3)	N1—N2	1.368 (2)
C1—H1	0.9300	N3—N4	1.368 (3)
			
N2^i^—Fe1—N2^ii^	180.00 (6)	C3—C2—H2	127.4
N2^i^—Fe1—N2	91.67 (10)	N2—C3—C2	110.30 (18)
N2^ii^—Fe1—N2	88.33 (10)	N2—C3—H3	124.9
N2^i^—Fe1—N2^iii^	88.33 (10)	C2—C3—H3	124.9
N2^ii^—Fe1—N2^iii^	91.67 (10)	N4—C4—C5	110.13 (13)
N2—Fe1—N2^iii^	180.00 (14)	N4—C4—H4	124.9
N2^i^—Fe1—N4^iii^	89.05 (7)	C5—C4—H4	124.9
N2^ii^—Fe1—N4^iii^	90.95 (7)	C6—C5—C4	104.89 (14)
N2—Fe1—N4^iii^	90.95 (7)	C6—C5—H5	127.6
N2^iii^—Fe1—N4^iii^	89.05 (7)	C4—C5—H5	127.6
N2^i^—Fe1—N4	90.95 (7)	N3—C6—C5	108.8 (2)
N2^ii^—Fe1—N4	89.05 (7)	N3—C6—H6	125.6
N2—Fe1—N4	89.05 (7)	C5—C6—H6	125.6
N2^iii^—Fe1—N4	90.95 (7)	C1—N1—N2	109.85 (16)
N4^iii^—Fe1—N4	180.00 (18)	C1—N1—B1	132.23 (18)
N1^ii^—B1—N1	108.4 (2)	N2—N1—B1	117.93 (17)
N1^ii^—B1—N3	106.87 (15)	C3—N2—N1	106.59 (16)
N1—B1—N3	106.87 (15)	C3—N2—Fe1	133.71 (14)
N1^ii^—B1—H1A	111.6 (9)	N1—N2—Fe1	119.67 (13)
N1—B1—H1A	111.6 (9)	C6—N3—N4	109.5 (2)
N3—B1—H1A	111.2 (17)	C6—N3—B1	131.8 (2)
N1—C1—C2	108.08 (18)	N4—N3—B1	118.6 (2)
N1—C1—H1	126.0	C4—N4—N3	106.6 (2)
C2—C1—H1	126.0	C4—N4—Fe1	134.45 (17)
C1—C2—C3	105.17 (18)	N3—N4—Fe1	118.91 (18)
C1—C2—H2	127.4		
			
N1—C1—C2—C3	0.4 (2)	N4^iii^—Fe1—N2—N1	−136.67 (14)
C1—C2—C3—N2	−0.2 (2)	N4—Fe1—N2—N1	43.33 (14)
N4—C4—C5—C6	0.0	C5—C6—N3—N4	0.0
C4—C5—C6—N3	0.0	C5—C6—N3—B1	180.0
C2—C1—N1—N2	−0.6 (2)	N1^ii^—B1—N3—C6	122.08 (15)
C2—C1—N1—B1	179.4 (2)	N1—B1—N3—C6	−122.08 (15)
N1^ii^—B1—N1—C1	−124.0 (2)	N1^ii^—B1—N3—N4	−57.92 (15)
N3—B1—N1—C1	121.1 (2)	N1—B1—N3—N4	57.92 (15)
N1^ii^—B1—N1—N2	55.9 (3)	C5—C4—N4—N3	0.0
N3—B1—N1—N2	−58.9 (2)	C5—C4—N4—Fe1	−180.0
C2—C3—N2—N1	−0.2 (2)	C6—N3—N4—C4	0.0
C2—C3—N2—Fe1	178.11 (14)	B1—N3—N4—C4	180.0
C1—N1—N2—C3	0.4 (2)	C6—N3—N4—Fe1	180.0
B1—N1—N2—C3	−179.50 (18)	B1—N3—N4—Fe1	0.0
C1—N1—N2—Fe1	−178.13 (13)	N2^i^—Fe1—N4—C4	44.17 (5)
B1—N1—N2—Fe1	1.9 (2)	N2^ii^—Fe1—N4—C4	−135.83 (5)
N2^i^—Fe1—N2—C3	−43.85 (17)	N2—Fe1—N4—C4	135.83 (5)
N2^ii^—Fe1—N2—C3	136.15 (17)	N2^iii^—Fe1—N4—C4	−44.17 (5)
N2^iii^—Fe1—N2—C3	39 (32)	N4^iii^—Fe1—N4—C4	0(100)
N4^iii^—Fe1—N2—C3	45.2 (2)	N2^i^—Fe1—N4—N3	−135.83 (5)
N4—Fe1—N2—C3	−134.8 (2)	N2^ii^—Fe1—N4—N3	44.17 (5)
N2^i^—Fe1—N2—N1	134.26 (16)	N2—Fe1—N4—N3	−44.17 (5)
N2^ii^—Fe1—N2—N1	−45.74 (16)	N2^iii^—Fe1—N4—N3	135.83 (5)
N2^iii^—Fe1—N2—N1	−143 (32)	N4^iii^—Fe1—N4—N3	180.00 (3)

Symmetry codes: (i) −*y*, −*x*, −*z*; (ii) *y*, *x*, *z*; (iii) −*x*, −*y*, −*z*.
